# Societal costs of fetal alcohol syndrome in Sweden

**DOI:** 10.1007/s10198-016-0811-4

**Published:** 2016-06-08

**Authors:** Lisa Ericson, Lennart Magnusson, Bo Hovstadius

**Affiliations:** 1 Department of Medicine and Optometry, eHealth Institute, Linnaeus University, 391 82 Kalmar, Sweden; 2 Department of Health and Caring Sciences, The Swedish Family Care Competence Centre, Linnaeus University, Kalmar, Sweden

**Keywords:** FAS, Direct costs, Indirect costs, Cost of illness, I10

## Abstract

**Objective:**

To estimate the annual societal cost of fetal alcohol syndrome (FAS) in Sweden, focusing on the secondary disabilities thought feasible to limit via early interventions.

**Methods:**

Prevalence-based cost-of-illness analysis of FAS in Sweden for 2014. Direct costs (societal support, special education, psychiatric disorders and alcohol/drug abuse) and indirect costs (reduced working capacity and informal caring), were included. The calculations were based on published Swedish studies, including a register-based follow-up study of adults with FAS, reports and databases, and experts.

**Results:**

The annual total societal cost of FAS was estimated at €76,000 per child (0–17 years) and €110,000 per adult (18–74 years), corresponding to €1.6 billion per year in the Swedish population using a prevalence of FAS of 0.2 %. The annual additional cost of FAS (difference between the FAS group and a comparison group) was estimated at €1.4 billion using a prevalence of 0.2 %. The major cost driver was the cost of societal support.

**Conclusions:**

The cost burden of FAS on the society is extensive, but likely to be underestimated. A reduction in the societal costs of FAS, both preventive and targeted interventions to children with FAS, should be prioritized. That is, the cost of early interventions such as placement in family homes or other forms of housing, and special education, represent unavoidable costs. However, these types of interventions are highly relevant to improve the individual’s quality of life and future prospects, and also, within a long-term perspective, to limit the societal costs and personal suffering.

## Introduction

Fetal alcohol syndrome (FAS) was first described in 1973 [1, 2] and is caused by maternal alcohol consumption during pregnancy. Alcohol is a teratogen that easily crosses the placenta and thus can have devastating effects on the developing embryo and fetus [3, 4]. The brain is particularly vulnerable to prenatal alcohol exposure [5] and the most profound effects are on brain development and on cognitive and behavioural outcomes [3, 4]. There is no safe “lower limit” for alcohol consumption during pregnancy and the absolute amount of alcohol that will not cause damage to the developing brain is not known. Prenatal exposure to alcohol can disrupt fetal brain development at any point during pregnancy [6] and the disturbances are more frequent and severe if the mother abuses alcohol throughout pregnancy [7, 8]. A recent Swedish study reported that relatively few women continue to drink alcohol during pregnancy. Nevertheless, 84 % of the women reported drinking in the year preceding pregnancy and most of these women continued to drink until pregnancy recognition [9].

The diagnosis of FAS has been expanded and refined and there are currently four widely used criteria. These diagnostic criteria have some differences, yet rely on anomalies in three distinct areas; pre- and/or postnatal growth deficiency, central nervous system dysfunction, and characteristic facial anomalies [10–13] (for review see [4]). Whether there also needs to be a confirmed maternal alcohol exposure varies between different diagnostic criteria [4]. Prenatal alcohol exposure causes a wide range of neuropsychological and behavioural disabilities and to identify all these different outcomes the non-diagnostic umbrella term “fetal alcohol spectrum disorders” (FASD), where the full-blown FAS is included, has come into use [4]. FAS is the only diagnosis defined according to the International Classification of Diseases (ICD-10; code Q86.0) [14].

FAS is recognized as the most common identifiable cause of acquired mental intellectual development disorder [3]. The true prevalence of FAS is not known. The latest prevalence study in Sweden was performed in the late 1970s and reported an incidence rate of fetal alcohol lesion of one per 300 deliveries of whom half had the complete FAS [15], corresponding to a prevalence of FAS of 0.2–0.3 % (or 19,000–29,000 individuals). Recent in-school studies estimated the prevalence of FAS at 0.2–0.9 % in the United States [16, 17] and 5.9–9.1 % in a South African community [18].

Brain dysfunction in children with FAS often leads to primary disabilities such as poor adaptive functioning, language deficits, attention difficulties and reasoning and memory deficits [6]. The neuropsychological and neurobehavioural problems often stem from early childhood, during adolescence and into adulthood [5]. Secondary disabilities that arise after birth as a result of the primary disabilities include psychiatric disorders, disrupted school and employment experiences, alcohol abuse/illicit drug use, trouble with the law and inappropriate sexual behaviour [6, 19]. A Swedish research group found that adults who were diagnosed with FAS in childhood revealed impaired psychosocial outcomes [20], decreased cognitive and executive functions and impaired social cognition [21]. Furthermore, individuals with FAS often have problems with their daily planning and living an independent life [3, 6].

However, adverse life outcomes in terms of secondary disabilities may be limited by protective factors such as receiving the diagnosis of FAS at an early age, placement in a stable environment and appropriate interventions for primary and secondary disabilities [6, 19]. In addition, children with FASD who were placed at an early age in a family home (formerly foster home), were seen to have a decreased risk of developing secondary disabilities [22]. Placement in a family home has also been suggested to lead to improved overall performance and a better quality of life for affected children in Sweden, although normalization does not occur [7, 8]. Special education and assistance in Swedish schools are also factors that are believed to limit the secondary disabilities [20].

Estimates of the economic impact of FAS/FASD demonstrate a significant cost burden on the individual, family and society. Unfortunately, health economic studies of FAS/FASD are sparse and the majority have been conducted in Canada and the United States [23]. Although these studies included many cost components, the total cost of prenatal alcohol exposure is underestimated [24]. Currently, to the best of our knowledge, there is a lack of cost calculations of FAS/FASD from a Swedish perspective.

The aim of this study was to perform a health economic calculation of the annual societal cost of FAS in Sweden, focusing on the secondary disabilities thought feasible to limit via early interventions. The present study is part of a major initiative in Sweden in the area of children as next of kin and was commissioned by the Swedish National Board of Health and Welfare. The study focuses on the economic cost of FAS in Sweden, in the light of relevant international studies.

## Methods

This study is a prevalence-based cost-of-illness analysis. Both the bottom-down approach, where costs for a defined subpopulation were estimated and then extrapolated to represent the entire population, and the top-down approach, where national frequencies and events formed the basis for estimates, was used [25, 26].

The present calculations are based on Swedish studies and Swedish cost data. Information that was otherwise inaccessible and/or unreliable was obtained from relevant experts such as a paediatrician, child psychiatrist, child welfare officer, care administrator, family members/relatives of individuals with FAS, researchers and staff at special accommodation facilities.

The cost of FAS was calculated as the annual total societal cost and the annual additional costs of FAS, based on the difference between the cost in the FAS group and a comparison group. The calculations are based on individuals up to the age of 64 years, and are also divided by age into children (0–17 years) and adults (18–64 years).

All costs were estimated in Swedish Crowns (SEK) according to 2014 prices and then converted to euros (€1.00 = SEK9.097) and, where applicable, the consumer price index was applied as the conversion factor.

### Study population

The proportion of individuals with the various secondary disabilities was adapted from a Swedish long-term follow-up study of psychosocial outcomes in adults with a verified diagnosis of FAS, including register-based anonymised data. The FAS group (*n* = 79; mean age 32 years) was composed of individuals who were all diagnosed with FAS at the Children’s Hospital in Gothenburg, Sweden, when they were infants or children. An additional comparison group (*n* = 3160) matched by age, gender, and place of birth was also included [20].

### Selection of secondary disabilities

Table 1 provides an overview of the secondary disabilities that appear in varying degrees in individuals with FAS and that serves as the basis for the present calculations. The selection of secondary disabilities is based on data with a significant difference between the FAS group and the comparison group, in the follow-up study conducted by Rangmar et al. [20].

**Table 1:** Overview of secondary disabilities in fetal alcohol syndrome (FAS) and description of the selection process in the health economic calculations

Secondary disability	Selection
Difficulties managing daily life	Societal support^a^ in terms of different forms of housing conditions
Problems at school	Special education (school for children with intellectual development disorders, primary school level)
Psychiatric disorder^b^	Average cost of depression, bipolar disorder, generalized anxiety disorder and schizophrenia
Alcohol/drug abuse	Alcohol abuse and/or illicit drug use
Suicidal ideation	No separate calculation
	Included in other cost references
Criminality	No separate calculation
	Cost included in alcohol/drug abuse
Inappropriate sexual behaviour	Not included
	Lack of data
Reduced working capacity	Productivity loss for individuals without employment
Mortality	No separate calculation
	Cost included in alcohol/drug abuse
Informal caring	Productivity loss for family member/relative/significant other

^a^Societal support is based on the proportion of individuals placed in state care (out-of-home care) and includes family care and residential care/orphanage (orphanages are no longer in use in Sweden, but were in operation when those in the study population were children) [20]

^b^The register extract included all diagnoses within the psychiatric chapter according to the ICD-9 classification system, except alcohol- and drug-related diagnoses [20]

### Direct costs of fetal alcohol syndrome

#### Societal support

In this study, the difficulties in managing daily life and living independently among individuals with FAS were classified as the need for societal support in terms of different forms of housing. The proportion of individuals with societal support was based on register data showing that significantly more individuals in the FAS group than in the comparison group had been placed in state care (81.0 vs 3.9 %) [20]. There was, however, no information available about the distribution between the different forms of housing conditions.

The cost among children (0–17 years) was calculated as a weighted average cost between family home and residential care (e.g. residential care homes for children and young people, in Sweden called HVB homes) [27], based on the assumption that 75 % of affected children grow up in a family home and 25 % in residential care. The cost among adults (18–64 years) was calculated as the mean cost of special accommodation according to two Swedish Acts; the Social Services Act (so-called SoL) and the Swedish Act concerning support and services for persons with certain functional impairments (so-called LSS) [28]. In the study by Rangmar et al. [20], some individuals had grown up at an orphanage, but since such living arrangements are no longer in use in Sweden this cost was not included in the weighted average cost.

#### Special education

Problems at school were based on the proportion of children who had attended school for children with special needs. A significantly higher percentage of children had received special education at primary school level in the FAS group than in the comparison group (25.3 vs 1.6 %) [20]. The cost of school for children with intellectual disabilities was retrieved from the Swedish National Agency for Education [29], which also included school transport and travel expenses compensation. There was no information about special needs teachers or personal assistance, thus these variables were not included in the calculations.

#### Psychiatric disorders

The prevalence of psychiatric disorders is based on data showing that a significantly higher percentage of individuals had been treated for a psychiatric disorder in the FAS group than in the comparison group (32.9 vs 4.7 %) [20]. The type of psychiatric disorder was not specified. The cost calculation for psychiatric disorders was delimited to adults (18–64 years) since there was no information about the psychiatric care needs among children with FAS. The cost of psychiatric disorders was adapted from a compilation of Swedish health economic studies [30] including depression [31], bipolar disorder [32], generalized anxiety disorder [30] and schizophrenia [33] in adults (≥18 years). Productivity loss was excluded from the estimation of psychiatric disorder cost, since this cost is included in the calculation of reduced working capacity (see below). Besides, there was no information as to whether individuals with a psychiatric diagnosis were in employment or not. Furthermore, the use of psychotropic drugs was not included since costs of pharmaceuticals were already included in the cost reference for psychiatric disorders [30] and alcohol/drug abuse [34], respectively. However, pharmaceuticals usually constitute a marginal portion of the total cost in health economic evaluations [31, 32].

#### Alcohol/drug abuse

The proportion of individuals who had been treated at hospital for alcohol/drug abuse was significantly higher in the FAS group than in the comparison group (12.7 vs 3.4 %) [20]. Since the abuse data included a rather small number of individuals, alcohol abuse and illicit drug use was merged. The cost of alcohol/drug abuse was adapted from an investigative assignment from the Swedish Government (the Swedish Abuse Investigation) including annual costs for different actors such as municipality, county council, legal system (state) and productivity loss [34]. Productivity loss was excluded from the estimation of alcohol/drug abuse since this cost is included in the calculation of reduced working capacity (see below). As above, there was no information as to whether individuals with alcohol/drug abuse were in paid employment or not.

### Indirect costs of fetal alcohol syndrome

#### Reduced working capacity

The cost of reduced working capacity was based on the proportion of individuals that were not employed (50.8 % in the FAS group and 14.7 % in the comparison group) [20]. The productivity loss was calculated as the salary loss, i.e. the labour cost of lost working time over one year, based on the salary for all-year full-time employees (20–64 years) [35]. Disability pension and/or social welfare payments were not included.

#### Informal caring

Being a parent/foster parent to a child with FAS involves extensive informal caring that most often impacts on the carer’s paid employment. The cost of informal caring was based on a parent that had to reduce their working time due to the child’s extensive care needs, estimated at 75 % part-time job when the child is <18 years old (personal communication, the FAS association).

In the FAS group, it was estimated that the proportion of individuals engaged in informal caring was 100 %. The corresponding proportion of informal caring in the Swedish population in individuals of working age (18–64 years) was estimated at 16 %. This calculation was based on a report on informal caring from the Swedish National Board of Health and Welfare [36] and population statistics from Statistics Sweden [35] (900,000/5,799,489 = 0.16).

As above, the productivity loss was calculated as the salary loss. In the FAS group, the lost working time for carers was estimated at 25 % when the child is 0–17 years old. Since there was no available corresponding estimate for the normal population, the same figure was used in the comparison group. Considering both the proportion of informal caring and the productivity loss, this gave figures of 25 % (1 × 0.25) in the FAS group and 4 % (0.16 × 0.25) in the comparison group.

## Results

The resource consumption was calculated as the annual mean direct and indirect costs for individuals with FAS and a comparison group, respectively (Table 2). The mean direct and indirect costs were also divided into the age categories children (0–17 years) and adults (18–64 years) in the FAS group (Table 3) and the comparison group (Table 4), respectively. Finally, the annual additional direct and indirect costs per individual with FAS were estimated as the difference between the FAS group and the comparison group (Table 5).

**Table 2:** The annual resource consumption per individual in the FAS group and the comparison group, respectively

		FAS group (*n* = 79)		Comparison group (*n* = 3160)	
Resources	Annual unit cost per individual (€)	Proportion of individuals (%)	Annual mean cost per individual (€)	Proportion of individuals (%)	Annual mean cost per individual (€)
Direct costs					
Societal support 0–17 years	60,043	81.0	48,635	3.9	2342
Societal support 18–64 years	77,549	81.0	62,815	3.9	3024
Special education^a^	53,745	25.3	13,598	1.6	860
Psychiatric disorder	8712	32.9	2866	4.7	409
Alcohol/drug abuse	126,466	12.7	16,061	3.4	4300
Indirect costs					
Reduced working capacity^a^	55,394	50.8	28,140	14.7	8143
Informal caring	55,394	25.0	13,849	4.0	2216

^a^ *n* = 75 in the FAS group and *n* = 2832 in the comparison group

**Table 3:** The annual mean cost (€) per individual in the FAS group in children (0–17 years) and adults (18–64 years), respectively

Resources	Annual mean cost per individual (€)	
	0–17 years	18–64 years
Direct costs		
Societal support	48,635	62,815
Special education	13,598	–
Psychiatric disorder	–	2866
Alcohol/drug abuse	–	16,061
Indirect costs		
Reduced working capacity	–	28,140
Informal caring	13,849	–
Total direct cost	62,233	81,743
Total indirect cost	13,849	28,140
Total cost	76,081	109,833

**Table 4:** The annual mean cost (€) per individual in the comparison group in children (0–17 years) and adults (18–64 years), respectively

Resources	Annual mean cost per individual (€)	
	0–17 years	18–64 years
Direct costs		
Societal support	2342	3024
Special education	860	–
Psychiatric disorder	–	409
Alcohol/drug abuse	–	4300
Indirect costs		
Reduced working capacity	–	8143
Informal caring	2216	–
Total direct cost	3202	7734
Total indirect cost	2216	8143
Total cost	5417	15,877

**Table 5:** The estimated annual mean additional direct and indirect costs (€) per individual in the FAS group in children (0–17 years) and adults (18–64 years), respectively

Resources	Annual mean additional cost per individual (€)	
	0–17 years	18–64 years
Direct costs		
Societal support	46,293	59,791
Special education	12,738	–
Psychiatric disorder	–	2457
Alcohol/drug abuse	–	11,761
Indirect costs		
Reduced working capacity	–	19,997
Informal caring	11,633	–
Total additional direct cost	59,031	74,009
Total additional indirect cost	11,633	19,997
Total additional cost	70,664	94,006

### Direct and indirect costs per individual

#### The annual cost in the FAS group and the comparison group

Table 2 shows the annual resource consumption per individual, i.e. type of resource (secondary disability), proportion of individuals with the respective secondary disability and cost, in the FAS group and the comparison group, respectively.

#### Costs divided by age group

Tables 3 and 4 show the annual mean direct and indirect cost per individual among children (0–17 years) and adults (18–64 years) in the FAS group (Table 3) and the comparison group (Table 4), respectively.

In children with FAS, societal support represents the highest cost (64 % of the total cost). Similarly, in adults with FAS, societal support constitutes the highest cost (57 %) followed by reduced working capacity (26 %).

In children in the comparison group, societal support represents the highest cost (43 % of the total cost), followed by informal caring (41 %). In adults, reduced working capacity constitutes the highest cost (51 %), followed by alcohol/drug abuse (27 %).

#### Additional costs of FAS

Table 5 shows the estimated annual direct and indirect additional costs, respectively, per individual with FAS. The additional cost was calculated as the difference between the individual cost in the FAS group and the comparison group, and is based on figures in Table 2.

The additional direct cost represented the highest proportion of the total additional cost both in children (84 %) and adults (79 %).

### Total societal costs

Based on the figures in Table 3 and adapting a prevalence of 0.2 %, the societal cost of FAS in Sweden was estimated at €1583 million per year, €297 million among children and €1286 million among adults. (With a prevalence of 0.2 % the estimated number of individuals with FAS in Sweden is about 4000 children aged 0–17 years and 12,000 adults aged 18–64 years).

Similarly, applying figures from Table 5 and a prevalence of 0.2 %, the additional societal cost of FAS in Sweden was estimated at €1385 million per year, €286 million among children and €1099 million among adults.

#### Sensitivity analyses

To assess the relative uncertainty in parts of the results, calculations with altered variables were performed. For example, there was a relative uncertainty in the proportion of adults with societal support and also in the mean cost level of societal support. This may have affected the calculations since societal support was the most cost driving factor both among children and adults with FAS (64 and 57 %, respectively, of the total cost).

It was assumed that the proportion of adults with FAS that need societal support was the same as the proportion of children with FAS with societal support (81 %). The cost level for adults with FAS was estimated as the mean cost of special accommodation according to SoL and LSS (Swedish Acts described in the Methods Section). It was assumed that 25 % of the children with FAS who need societal support grow up in HVB homes and 75 % in family homes. An adjustment in the weighted average between HVB home and family home only gives a marginal change in the societal cost. However, altering the proportion of adults living in special accommodation from 81 to 50 % decreases the annual total societal cost from €1583 to €1308 million and the annual additional cost from €1385 to €1099 million.

In the extrapolation of cost data from an individual level to a population level using an estimated prevalence also involves an element of uncertainty. The estimated prevalence of FAS of 0.2 %, which is in the lower part of the interval in Olegård et al. [15], is low compared with international studies. From an international perspective, using a low prevalence of 0.1 % the annual total societal cost and annual additional cost would be halved (€791 and €693 million, respectively). Instead, using a higher prevalence of 0.3 %, results in a total societal cost of about €2418 million per year and an additional cost of FAS of about €2089 million per year.

## Discussion

To the best of our knowledge this is the first health economic study of the societal cost of FAS in Sweden. The calculations are based on the secondary disabilities that develop in varying degrees in individuals with FAS. The occurrence of secondary disabilities was adapted from a Swedish register-based long-term follow-up study of psychosocial outcomes in adults with a verified diagnosis of FAS [20]. The annual total societal cost of FAS was estimated at about €76,000 per child and €110,000 per adult, and the major proportion was represented by direct costs (82 and 74 %, respectively). Applying a prevalence of FAS of 0.2 % (corresponding to ~19,000 individuals in the entire population), the total societal cost was estimated at €1.6 billion per year in the Swedish population. This assumption is based on a previous Swedish study [15] and is deemed low compared with international studies from e.g. the United States (0.2–0.9 %), but within the interval [16, 17]. To the best of our knowledge, there are no additional published prevalence studies in Sweden. The major cost driver was the cost of societal support, both in children and adults with FAS. The direct costs represented the highest proportion of the total cost both in children and in adults with FAS, whereas there were only minor differences in the proportion of direct and indirect costs in the comparison group.

A complicating factor in this study was the actual number of children and adults with alcohol damage to date in the society. Often adverse life outcomes are not restricted to those with or without the classical facial features of FAS or to those with or without mental retardation [19]. Not all children born with alcohol damage have the typical external features (facial anomalies) of FAS, but instead they have disabilities that are often difficult to diagnose, leading to an estimated number of unknown cases [13].

The secondary disabilities often lead to major societal costs and may be limited by early interventions (from the society). Protective factors helping children with FAS avoid adverse life outcomes include receiving the diagnosis of FAS at an early age, placement in a stable environment and appropriate interventions for primary and secondary disabilities [6, 19]. International cost studies have shown that prenatal alcohol exposure is associated with a major health care and cost burden on the society [37–40]. For example, the total direct cost of FAS in Canada in 2008–2009, was estimated at about €5.3 million (authors’ recalculation from Canadian dollars to euros at 2014 cost level) [37]. In another Canadian study, the total adjusted annual costs associated with FASD were estimated at ~€16,000 per individual [39]. Furthermore, the annual cost of care among children with FASD ranged from €43.3 to €148.7 million [38]. It is, however, difficult to compare the results from different cost studies, since the methodology and population often varies between the studies.

In this study, societal support represented the highest portion of the total cost based on ~80 % of children growing up in some form of special accommodation, but with unknown distribution between family home and residential care. It was, however, assumed that the majority of children with FAS grow up in a family home, which was also reported in a Swedish follow-up study of children of alcoholic mothers [7, 8] where early placement in a family home was suggested to lead to improved performance and enhanced quality of life [7]. Similarly, early placement of children with FASD in a family home was able to limit disabilities, such as socio-emotional problems (including neuropsychological problems) [22]. In contrast, a further Swedish long-term follow-up study, reported that children placed in out-of-home care showed a higher risk of psychiatric problems, criminality and low educational levels, and that these problems were higher in children placed for their behavioural problems [41]. The prognosis of individuals with FAS is in some cases relatively optimistic, since a child with FAS in Sweden at risk of growing up in an inappropriate environment, with e.g. abuse, is routinely taken care of and generally receives correct care early [20], compared with children taken care of during adolescence due to their own behavioural problems [41]. Placement in a family home can thus be seen as an unavoidable cost, yet an important intervention commonly preventing serious problems.

Due to their limited basic intelligence, a high proportion of children with FAS/FASD attend school for children with special educational needs [7, 42]. Since many of these children also have behavioural difficulties, even those children with normal intelligence often end up in special school [42]. However, some children attend normal school with extra support, such as a special needs teacher and/or personal assistance. In the study by Rangmar and colleagues, a quarter of children with FAS had attended a special needs school (primary school level) and only about 5 % had secured a postsecondary qualification [20]. In Sweden, the cost of special school is approximately five times higher than normal school [29]. Additional costs associated with normal school attendance includes, for example, extra support, but the extent of such support within the FAS population is not known. However, the individuals with FAS who had attended normal school most likely had had access to personal assistance at school, at least for a limited period. It can be argued that, in the long term, it should not solely be a discussion about the costs, since the opportunity of attending special school may result in individuals with FAS being able to work, which is positive both for the individual (quality of life) concerned and from a societal perspective. The cost of special school is thus difficult to avoid since adapted schooling is a necessary early intervention that may prevent other costs later in life.

Mental illness is relatively common among individuals with FAS/FASD [20, 21, 43] and the problems often persist throughout life [20, 42]. The cost of psychiatric disorders among adults with FAS was only a minor part of the total societal cost. This can partly be explained by the exclusion of productivity loss from the cost of psychiatric disorders, since productivity loss was already included in the calculation of reduced working capacity. In addition, the calculation of the cost of psychiatric disorders was based on four diagnoses (depression, bipolar disorder, generalized anxiety disorder and schizophrenia) [30], which were deemed to be representative of reflecting mental illness. The cost of psychiatric disorders was only estimated for adults, but the majority of these individuals were probably also treated during childhood and/or adolescence. Nevertheless, information about the need for psychiatric care among children/adolescents was lacking.

Individuals with FAS are at increased risk of developing problems with alcohol and/or illicit drugs [19]. However, this was not so apparent in the long-term study by Rangmar et al. [20]. The societal cost of alcohol/drug abuse was thus not so high, although not negligible, as it constituted 15 % of the annual total societal cost among adults with FAS. Alcohol abuse and/or illicit drug use often leads to criminality and individuals with FAS may easily find themselves falling into trouble with the law [6]. Canadian data highlighted that youths with FASD are 19 times more likely to be in prison than youths without FASD [44]. Comparable Swedish data is not available. Besides, the cost of criminality was already included in the cost reference for alcohol/drug abuse [34].

The working capacity of individuals with FAS is often reduced, resulting in unemployment, disability pension and/or social welfare payments. Half of the individuals with FAS in the Swedish study were unemployed [20] resulting in a productivity loss corresponding to a quarter of the total societal cost. At the same time, half of the individuals with FAS were employed; nevertheless, the disposable income indicated lower-paid jobs [20]. Even though these jobs were supplemented by welfare benefits, it can be argued that it is positive from a societal perspective to be self-sufficient.

The caring support to individuals with FAS from family members/relatives is of high intensity, due to the disabilities that commonly develop in FAS as a result of brain damage caused by prenatal alcohol exposure. The productivity loss among parents/foster parents, who often have to take on board considerable responsibility as informal carers, is calculated as almost one fifth (18 %) of the societal cost among children with FAS (based on informal caring by one parent per family). This estimation was based on 25 % reduced working time (in both groups) when the child is below 18 years of age, with the distinction that all individuals in the FAS group need informal caring compared with around one fifth in the general population. This can be compared with a Swedish study about the burden of informal caring of patients with psychoses, showing that informal carers spent 22.5 h per week on caring activities and about 14 % of their gross income on care-related activities [45].

### Limitations and judgement of costs

The cost levels of the resources included in the calculations, and the proportion of individuals, are considered valid and based on sound published data. Nevertheless, sensitivity analyses were performed, including the major cost-driving variables, societal support and prevalence, respectively. Applying a lower proportion of adults with FAS receiving societal support (50 % instead of 81 %), resulted in about 20 % reduced annual total societal cost and additional societal cost, respectively. However, such an assumption should be followed by various assumptions of other supplemental support for adults with FAS, such as the cost of daily activities, which in part would compensate for the reduced societal cost.

Variations in the prevalence of FAS had even greater impact on the cost. There is probably a higher prevalence than the 0.2 % used in the present calculations that is closer to the true prevalence. Prevalence figures corresponding to international levels would give multiple times higher costs. It should be noted that the calculations only cover the estimated societal costs for the relatively narrow diagnosis of FAS. The prevalence of FASD is considerably higher [16, 18, 46] and even though some of these studies were based on specific populations/regions they give an indication of the magnitude of the problems following prenatal alcohol exposure. Altogether, this means that, most probably, there is a considerable underestimation of the total societal cost of FAS/FASD.

The starting point of this study was that FAS had already been diagnosed, and costs associated with the diagnostic investigation were thus not included. However, the diagnostic process of FAS includes a comprehensive assessment of the individual and is thus associated with additional costs [47]. In this study, only secondary disabilities were included in the calculations. Intangible costs such as pain and suffering were not included, as these costs are generally difficult to measure and value, nor were costs for premature mortality or quality of life included, because of lack of data. There were probably also other limitations that could have affected the results, such as insufficient basic data, risk of double counting, or concerned variables that were not part of the assignment. For example, disability pension and/or social welfare payments were not included in the calculations; however, for individual Swedish municipalities the cost of social welfare payments is a substantial factor in the local budget.

### Methods discussion

The calculations reflect a typical case of FAS. As all individuals probably do not develop all secondary disabilities, the calculations were based on the frequency of secondary disabilities in a specific study population. This study population comprised a regional FAS population [20]. That is, a regional prevalence of FAS was extrapolated to a national level, meaning the potential uncertainty in the data was scaled up. The ideal situation would have been to base the calculations on a larger population and/or several studies. However, the present calculations are considered to be well documented. Besides, information about these individuals was retrieved from national registries covering the entire Swedish population. These registers offer unique opportunities for research (follow-up studies), particularly as the information is based on unique personal identification numbers. The regional variation in the prevalence of FAS seen in Swedish registers (Swedish National Board of Health and Welfare, register extract) more likely reflects the level of knowledge in the county councils as opposed to the prevalence of alcohol damage. However, it cannot be excluded that it also reflects actual regional variations in the prevalence of FAS.

Both the annual total societal cost of FAS and the annual additional cost burden of FAS on the society, that is the difference between the FAS group and the comparison group, were calculated. Since the calculations included a comparison factor it might not be classified as a strict, but rather an “extended”, cost-of-illness analysis. Anyhow, this approach is part of the method we used for estimating the societal cost of FAS in Sweden.

Calculations are based on published cost estimates that are performed in several steps, either with a bottom-up or a top-down approach. In the present study a bottom-up approach was used, since there were no published top-down data for the studied population (FAS). In bottom-up analyses, the costs may be overestimated due to, among other things, the risk of double counting. However, by excluding such costs that were already included in the published cost data, the risk of double counting in the present study was reduced.

### Conclusions and future work

The cost burden of FAS on the society is extensive. The majority of these costs relate to different types of societal support during an individual’s lifetime. To decrease the societal costs of FAS, both preventive interventions, to minimize the risk of prenatal alcohol damage from arising, and targeted interventions in children with FAS, should be prioritized. One key factor in limiting secondary disabilities in individuals with FAS is early assessment and diagnosis, helping the children to receive the right support from the beginning. In that way their school attendance can be facilitated through extra support, which in turn may provide individuals with FAS the opportunity to secure paid work. In turn, this leads to lower societal costs both by a reduced need for societal support and by individuals being self-sufficient.

In conclusion, the placement costs for children with FAS in family homes or other forms of housing and the special education costs both represent unavoidable costs, since such early interventions can be key factors limiting the secondary disabilities that often develop in individuals with FAS. Thus, these types of interventions are highly important to improve the individual’s quality of life and future prospects, and also to limit both the societal costs and personal suffering in the long-term perspective.

The prerequisite for early, targeted interventions is early assessment and diagnosis of FAS/FASD. Clearly, there is a need for gathered competence of FAS/FASD in, e.g., special resource centres, including medical, psychosocial and pedagogical support of the child in the home environment and at school. In the preventive work, it is important to target all women of fertile age and engage in targeted public health initiatives that disseminate relevant information about the teratogenic effects (birth defects, malformations of the embryo and fetus) of alcohol consumption.

It is hoped that this Swedish study will help fill part of the current knowledge gap and be used as a foundation to clarify the major economic burden of FAS on the society and to provide appropriate timely support. Nevertheless, the area is highly complex and further studies are needed to gain a comprehensive view of the economic situation.
